# Identification and validation of core genes for type 2 diabetes mellitus by integrated analysis of single-cell and bulk RNA-sequencing

**DOI:** 10.1186/s40001-023-01321-1

**Published:** 2023-09-12

**Authors:** Tingting Yang, Chaoying Yan, Lan Yang, Jialu Tan, Shiqiu Jiang, Juan Hu, Wei Gao, Qiang Wang, Yansong Li

**Affiliations:** 1 Department of Anesthesiology & Center for Brain Science, The First Affiliated Hospital of Xi’an Jiaotong University, Xi’an, 710061 Shaanxi China; 2 Shaanxi University of Chinese Medicine, Xianyang, Shaanxi China

**Keywords:** Type 2 diabetes mellitus, Single-cell sequencing, Traditional transcriptome sequencing, *CDKN1C*, *DLK1*

## Abstract

**Background:**

The exact mechanisms of type 2 diabetes mellitus (T2DM) remain largely unknown. We intended to authenticate critical genes linked to T2DM progression by tandem single-cell sequencing and general transcriptome sequencing data.

**Methods:**

T2DM single-cell RNA-sequencing data were submitted by the Gene Expression Omnibus (GEO) database and ArrayExpress (EBI), from which gene expression matrices were retrieved. The common cell clusters and representative marker genes were ascertained by principal component analysis (PCA), t-distributed stochastic neighbor embedding (t-SNE), CellMarker, and FindMarkers in two datasets (GSE86469 and GSE81608). T2DM-related differentially expressed marker genes were defined by intersection analysis of marker genes and GSE86468-differentially expressed genes. Receiver operating characteristic (ROC) curves were utilized to assign representative marker genes with diagnostic values by GSE86468, GSE29226 and external validation GSE29221, and their prospective target compounds were forecasted by PubChem. Besides, the R package clusterProfiler-based functional annotation was designed to unveil the intrinsic mechanisms of the target genes. At last, western blot was used to validate the alternation of *CDKN1C* and *DLK1* expression in primary pancreatic islet cells cultured with or without 30mM glucose.

**Results:**

Three common cell clusters were authenticated in two independent T2DM single-cell sequencing data, covering neurons, epithelial cells, and smooth muscle cells. Functional ensemble analysis disclosed an intimate association of these cell clusters with peptide/insulin secretion and pancreatic development. Pseudo-temporal trajectory analysis indicated that almost all epithelial and smooth muscle cells were of neuron origin. We characterized *CDKN1C* and *DLK1*, which were notably upregulated in T2DM samples, with satisfactory availability in recognizing three representative marker genes in non-diabetic and T2DM samples, and they were also robustly interlinked with the clinical characteristics of patients. Western blot also demonstrated that, compared with control group, the expression of *CDKN1C* and *DLK1* were increased in primary pancreatic islet cells cultured with 30 mM glucose for 48 h. Additionally, PubChem projected 11 and 21 potential compounds for *CDKN1C* and *DLK1*, respectively.

**Conclusion:**

It is desirable that the emergence of the 2 critical genes indicated (*CDKN1C* and *DLK1*) could be catalysts for the investigation of the mechanisms of T2DM progression and the exploitation of innovative therapies.

**Supplementary Information:**

The online version contains supplementary material available at 10.1186/s40001-023-01321-1.

## Introduction

Diabetes, characterized by hyperglycemia caused by absolute or relative insulin deficiency, is a major contributing factor to atherosclerosis, coronary heart disease and premature death [1]. With a significant increase in the global prevalence of diabetes, the population of diabetics is estimated to 642 million by 2040 [2], at an estimated annual health care cost of $825 billion [3], making diabetes and its complications important public health issues. In contrast to T1 diabetes, the major subtype of diabetes is T2 diabetes mellitus (T2DM), also known as non-insulin-dependent diabetes, accounting for more than 90% of diabetics [4]. Although extensive research has been done into the pathogenesis of T2DM, the exact mechanism remains largely unknown. Hence, it is imperative to explore its possible specific mechanism and develop corresponding prevention and treatment strategies.

Pancreatic islet cells are critical for maintaining normal blood glucose levels, as damage to pancreatic islet cells can lead to the development and progression of diabetes. With the rapid development of high throughput second generation RNA sequencing (RNA-Seq), transcriptomic analysis of pancreatic islet provides an accurate approach to identify biomarkers in the pancreas of diabetics. However, bulk RNA analysis can only deliver the mean abundance for each gene. Given that islet cell composition is highly variable even among non-diabetic controls [5], the high heterogeneity of individual cells is masked, leading to absence and failure of studies on potential therapeutic target [6]. Since the advent of single-cell RNA sequencing (scRNA-seq) in 2009, which expanded our knowledge about biological systems [7], a dozen publication have reported results of scRNA-seq, providing insights into the heterogeneity of islet cells. Nevertheless, scRNA-seq is not yet possible to detect low-abundance transcripts due to limited efficiency of initial mRNA capture and cDNA conversion, which may lead to loss of valuable information and increased sampling errors [6]. Therefore, a combination of bulk RNA-seq with scRNA-seq analysis may be a proper option for identifying new diabetes therapeutic targets.

In this study, *CDKN1C* and *DLK1* were identified by intersected analysis of scRNA-seq datasets with bulk RNA-seq datasets, their alternation was verified in primary pancreatic islet cells cultured in high glucose. Besides, we forecasted the potential compounds targeting *CDKN1C* and *DLK1* in T2DM, providing a theoretical basis for future studies on the mechanisms of T2DM progression and the exploitation of innovative therapies in future.

## Results

### The single-cell landscape of human non-diabetic and T2DM

**Fig. 1:**
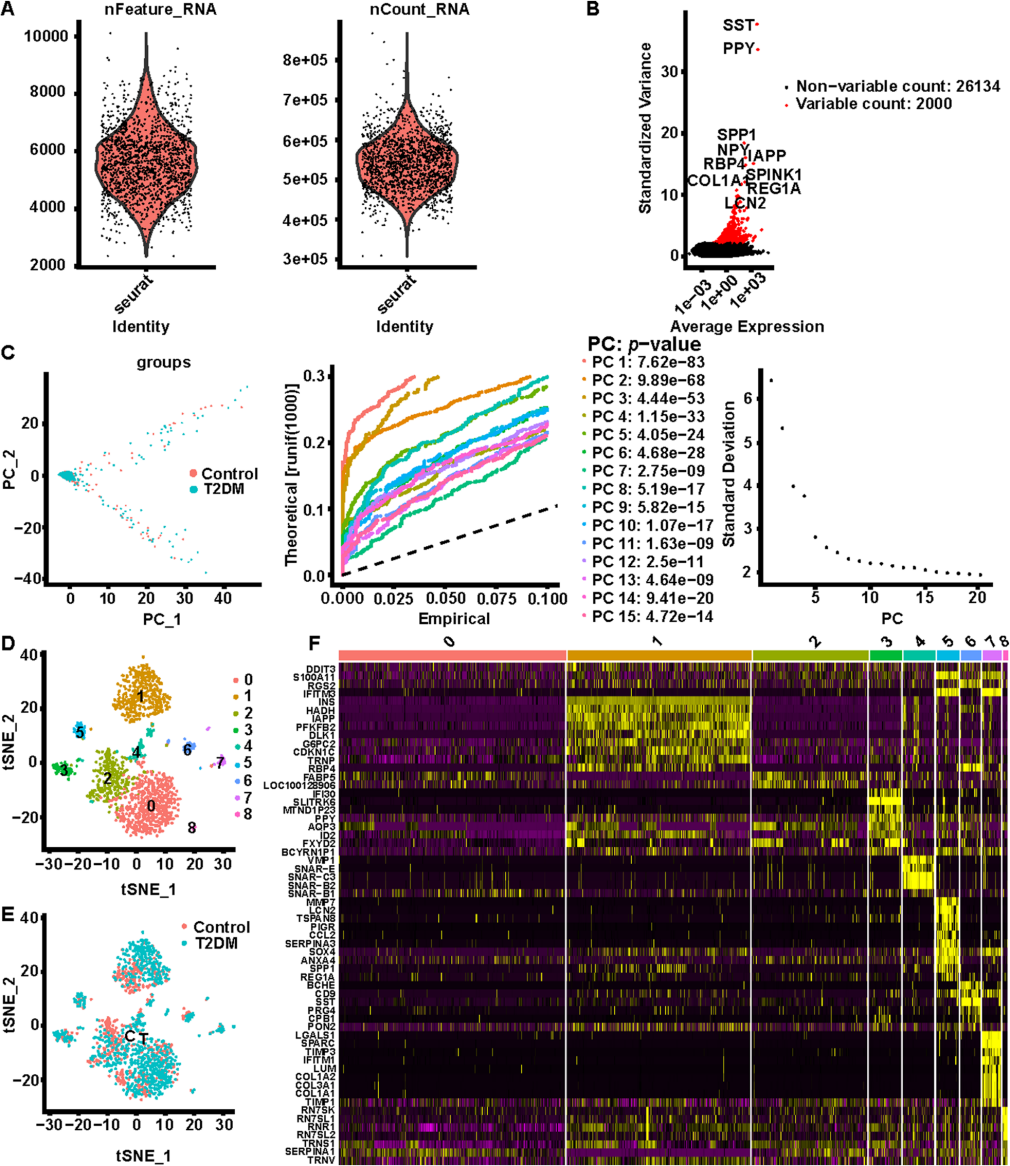
Cell processing of scRNA-seq Dataset GSE81608. **A** Number of genes per cell (nFeature_RNA) and number of unique molecular identifiers (UMIs) per cell (nCount_RNA) for scRNA-seq data. **B** The top 2000 variable DEGs between cells are marked in red, and the top 10 variable DEGs are labeled. **C** Identification of 15 principal components (PCs) with significant differences (*p* < 0.05), where we observe an ‘elbow’ around PC6-8, suggesting that the majority of true signal is captured in the first 8 PCs. **D** Identification of 9 cell clusters by performing T-distributed stochastic neighbor embedding (t-SNE). **E** Sample sources of each cluster. **F** Heatmap displaying top 60 marker genes in 9 clusters

**Fig. 2:**
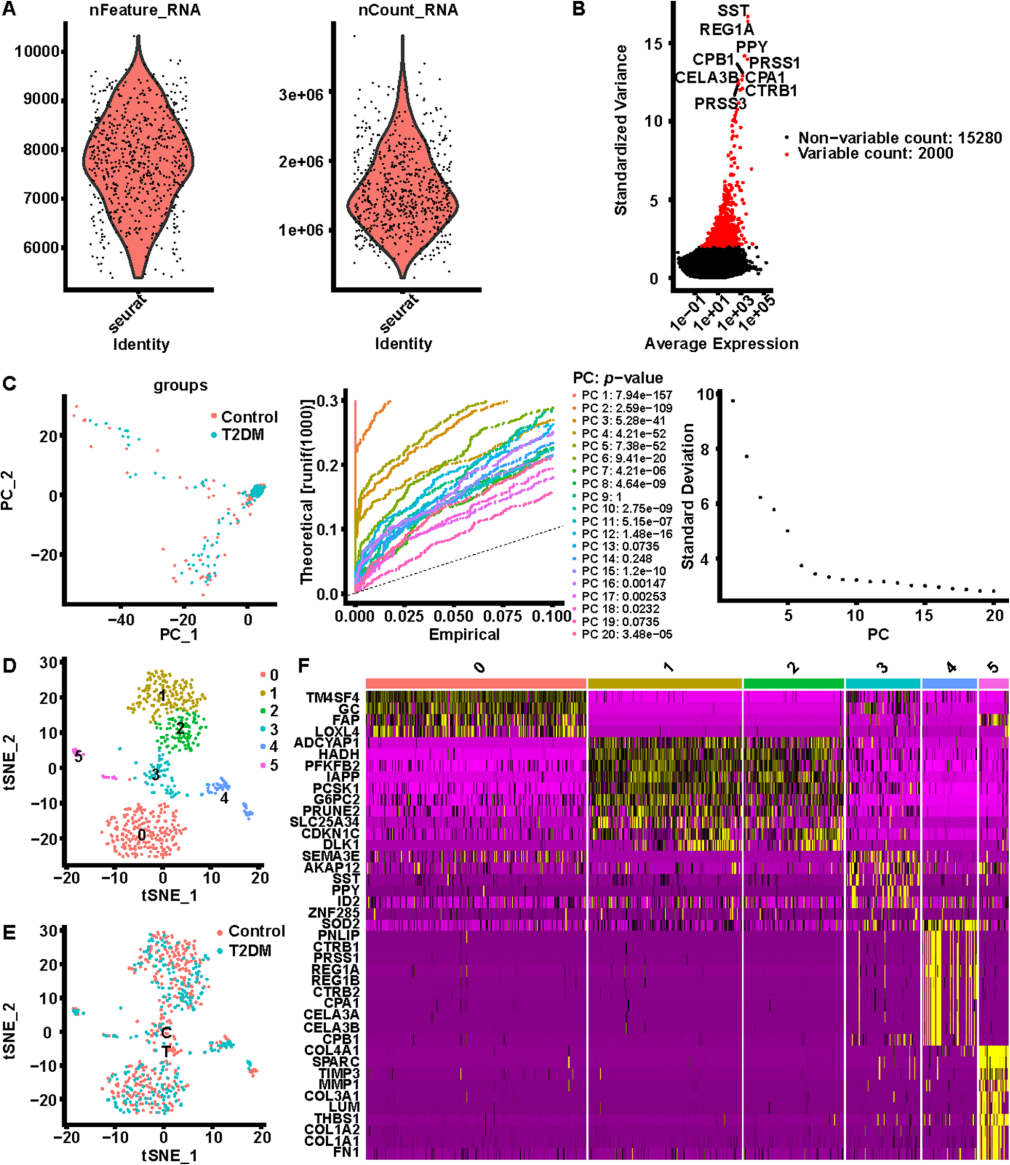
Cell processing of scRNA-seq dataset GSE86469. **A** Number of genes per cell (nFeature_RNA) and Number of UMIs per cell (nCount_RNA) for scRNA-seq data. **B** The top 2000 variable DEGs between cells are marked in red, and the top 10 variable DEGs are labeled. **C** Identification of 20 principal components (PCs) with significant differences (*p* < 0.05), where we observe an ‘elbow’ around PC6-8, suggesting that the majority of true signal is captured in the first 8 PCs. **D** Identification of 6 cell clusters by performing t-distributed stochastic neighbor embedding (t-SNE). **E** Sample sources of each cluster. **F** Heatmap displaying the top 40 marker genes in the 6 clusters

A total of 17 non-diabetic and 8 T2DM patient samples from the GSE81608 and GSE86469 datasets were covered in this study. Among them, 651 single cells were from non-diabetic (*n* = 12), while 949 were cells of T2DM (*n* = 5) origin in the GSE81608 dataset; in the GSE86469 dataset, 380 and 258 cells were extracted from non-diabetic (*n* = 5) and T2DM (*n* = 3) samples, respectively. After elimination of low-quality cells, a total of 2130 cells were finally included in the analysis, namely 622 cells in GSE81608-non-diabetic, 870 cells in GSE81608-T2DM (Fig. 1A), 380 cells in GSE86469-non-diabetic, and 258 cells in GSE86469-T2DM (Fig. 2A). PCA dimensionality reduction based on highly variable genes (k = 2000; Fig. 1B and 2B) was then performed for all cells in both datasets, respectively. PCA based on highly variable genes for all cells in each dataset (k = 2000; Figs. 1B and 2B) were then applied to affirm the available principal components (PCs) based on *P* < 0.05. Through the combinations of resolutions and PC value for the cluster identification, 15 PCs were selected in the GSE81608 dataset (Fig. 1C and Additional file 1: Fig. S1A), and 20 PCs were elected in the GSE86469 dataset (Fig. 2C and Additional file 1: Fig. S1B).

Then, the cells in GSE81608 were divided into 9 individual clusters (C0-C8; Fig. 1D) with the tSNE algorithm, and analogously, the cells in the GSE86469 dataset were partitioned into six primary cell lineages (C0-C5; Fig. 2D). Besides, Figs. 1E and 2E also illustrated the sample sources of each cluster in both datasets. Subsequently, 306 marker genes were characterized in the GSE81608 dataset-9 clusters by the FindAllMarkers function (Fig. 1F; Additional file 1: Table S1). A total of 649 marker genes from all 6 clusters (GSE86469 dataset) were authenticated (Fig. 2F; Additional file 1: Table S2).

## Phenotyping of human non-diabetic and T2DM cells

**Fig. 3:**
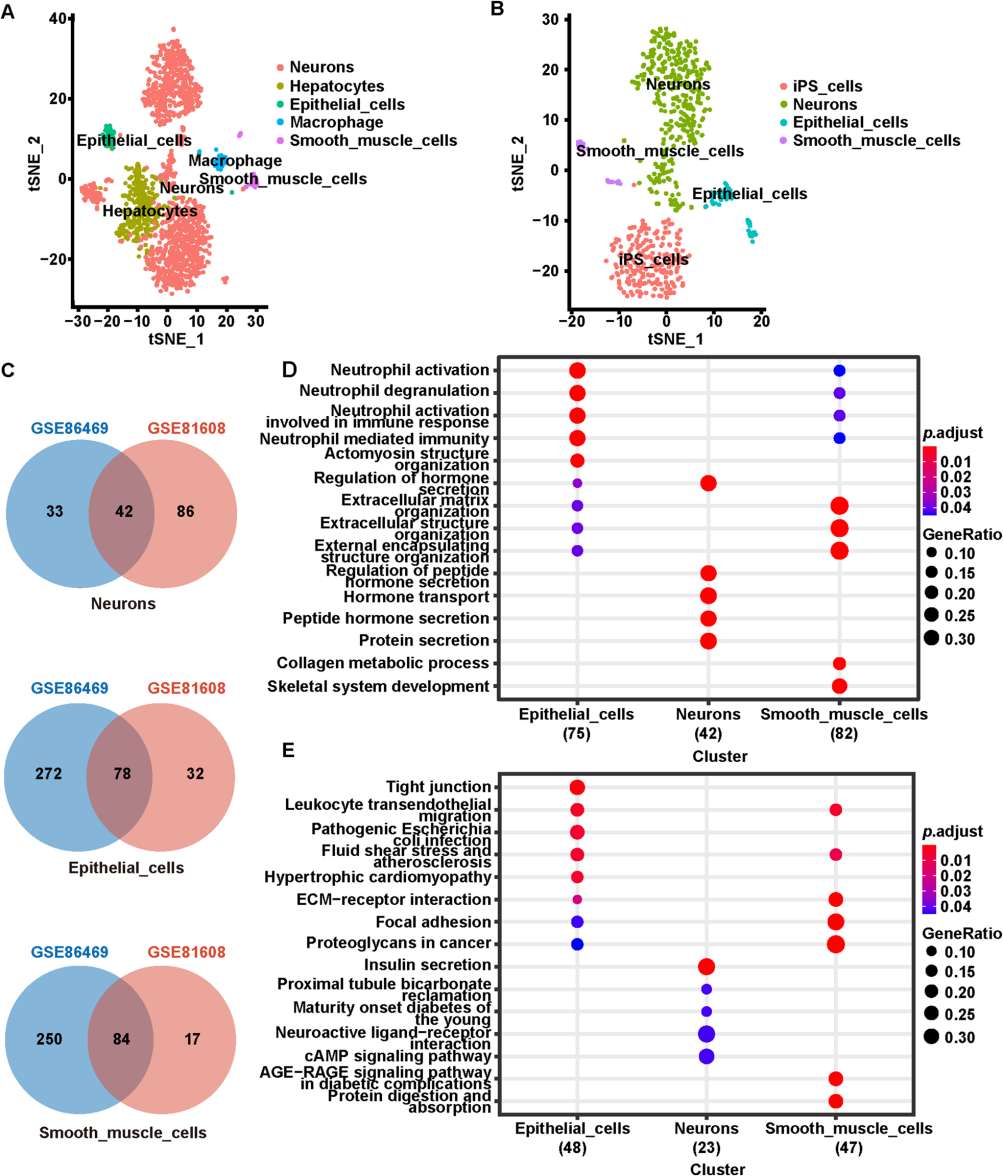
Cell annotation and latent mechanism of cell clusters in non-diabetic and T2DM. **A** Distribution of the cell subpopulations in the GSE81608. **B** Distribution of the cell subpopulations in the GSE86469. **C** Identification of identical marker genes in neuron, epithelial cell and smooth muscle cell cluster from GSE81608 and GSE86469 datasets utilizing cross-tabulation analysis. **D** BP categories of GO enrichment analysis of marker genes in neuron, epithelial cell and smooth muscle cell cluster. **E** KEGG enrichment analysis of marker genes in neuron, epithelial cell and smooth muscle cell cluster

Cell populations were annotated in the CellMarker database with the R package singl based on the expression patterns of the authenticated marker genes. In the GSE81608 dataset (Fig. 3A), C0, C1, C3, C4, and C8 were labeled as Neurons, C2 as Hepatocytes, C5 as Epithelial cells, C6 as Macrophage, and C7 as Smooth muscle cells. The six cell clusters from the GSE86469 dataset were illustrated as iPS cells (C0), neurons (C1, 2, and 3), epithelial cells (C4), and smooth muscle cells (C5) (Fig. 3B). Collectively, neurons, epithelial cells, and smooth muscle cells were the cell clusters annotated in both datasets, implying that they may potentially feature an essential role in non-diabetic and T2DM. Additionally, the cellular provenance of the three common cell clusters from both datasets is tabulated in Table 1.

**Table 1:** Cellular provenance of three common cell clusters

	Neurons		Smooth_muscle_cells		Epithelial_cells		All
	Non-diabetic	T2D	Non-diabetic	T2D	Non-diabetic	T2D	
GSE86469	214	119	12	18	27	2929	419
GSE81608	451	675	17	7	87	134	1371

## The latent mechanism of common cell clusters in non-diabetic and T2DM

Cross-tabulation analysis was utilized to pinpoint identical marker genes in each common cell cluster from the GSE81608 and GSE86469 datasets. The results are presented in Fig. 3C. In the two datasets, there were 42 common-marker genes between neurons (noted as neurons’ marker genes; Additional file 1: Table S3), a total of 78 epithelial cells’ marker genes (Additional file 1: Table S4), and 84 smooth muscle cells’ marker genes (Additional file 1: Table S5).

Thereafter, functional annotation was performed against these marker genes to enlighten the utility of the common cell cluster in non-diabetes and T2DM. These genes contained a total of 554 BP terms, 58 CC terms, and 68 MF terms in the GO system (Additional file 1: Table S6). This study primarily concentrated on the results of BP categories (Fig. 3D). Specifically, neurons’ marker genes were mainly linked to T2DM-related processes, such as peptide/peptide hormone secretion, insulin secretion, and carbohydrate/sugar metabolism. Epithelial cells’ marker genes were associated with neutrophil activation, degranulation and their mediated immunity; predictably, epidermal/epithelial differentiation/migration/apoptosis processes were notably enriched; moreover, these genes were also closely affiliated with wound healing, hemostasis/coagulation, vascular development, and lipid localization/translocation. Smooth muscle cells’ marker genes were predominantly enriched in the extracellular matrix and structural tissues; consistently, they were also tightly integrated with wound healing, hemostasis/coagulation, and vascular development; strikingly, pancreatic development and B-type pancreatic cell proliferation terms were distinctly enriched; meanwhile, smooth muscle cell/epithelial cell migration and neuronal death/projection expansion were also detected. KEGG enrichment analysis (Fig. 3E; Additional file 1: Table S7) indicated that neurons’ marker genes were participating in insulin secretion, glucagon signaling pathway, and maturity-onset diabetes in young adults; epithelial cells’ marker genes and smooth muscle cells’ marker genes were strongly coupled to the most common vascular outcome of T2DM, coronary heart disease (CHD) [8], especially the epithelial cells' marker genes, which were linked to multiple CHD-related pathways (hypertrophic and dilated cardiomyopathy, atherosclerosis, arrhythmogenic right ventricular cardiomyopathy). Additionally, marker genes of smooth muscle cells were cited in the pathway of AGE-RAGE signaling in diabetic complications.

## Pinpointing of aberrantly expressed marker genes in the common cell clusters

**Fig. 4:**
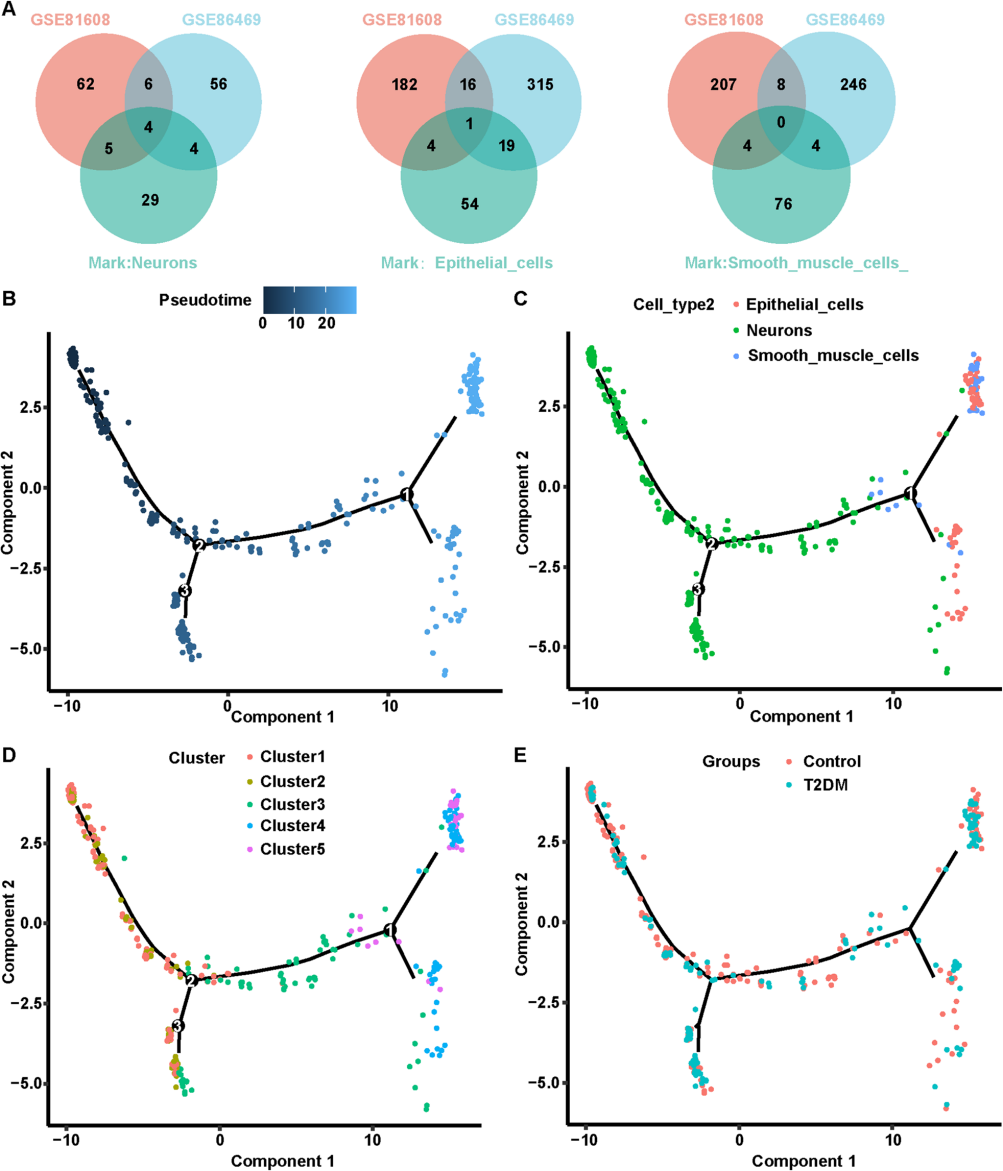
Analysis of marker genes and pseudo-time trajectory analysis of the common cells. **A** Robust DEMGs analysis in cell clusters of neurons, epithelial cells and smooth muscle cells by cross-tabulation. **B** Different cells in the GSE86469 dataset were ordered along trajectories to construct a pseudo-time axis. The darker the color, the earlier the cell progression begins. **C** Cell annotations in trajectory analysis. **D** Distribution of 5 sub-clusters in trajectory. **E** Distribution of non-diabetic or T2DM sample cells in trajectory

Next, the FindMarkers function (R package Seurat) was employed to analyze DEGs between non-diabetic and T2DM samples in the common cell cluster in GSE81608 (Additional file 1: Table S8) and GSE86469 (Additional file 1: Table S9) datasets. Then, robust DEMGs in neurons, epithelial cells, and smooth muscle cells cell clusters were investigated separately by using cross-tabulation analysis. As depicted in Fig. 4A, *TTR*, *SST*, *CDKN1C*, and *DLK1* were DEMGs in the neurons cluster; *SDC4* was a DE-marker gene in the epithelial cells cluster; no robust DE-marker gene was confirmed in the smooth muscle cells cluster.

## Pseudo-time trajectory analysis of the common cells

To further explore the landscape of neurons, epithelial and smooth muscle cells in the non-diabetic and T2DM microenvironments, the differentiation trajectories of neurons, epithelial cells, and smooth muscle cells from both sources (non-diabetic and T2DM) were mimicked in the GSE86469 dataset.

Genes with abnormal expression in neurons, epithelial cells, and smooth muscle cells populations were authenticated between non-diabetic and T2DM samples. A dendrogram of differentiation trajectories of the 3 cell lineages through these genes was then constructed using the R package Monocle (Fig. 4B). Concerning cell typing, the initiation of the branches and nodes 2 and 3 consisted of neurons, while the other two branches (at node 1) mainly consisted of epithelial and smooth muscle cells and a few neurons (Fig. 4C). From the perspective of the original clusters of cells (cell cluster name not labeled), the initiation segment of differentiation mainly contained C1 and C2 cells, the middle segment was composed of C3, and the end segment contained all C4 and C5 and a few C3 cells (Fig. 4D). In terms of cell origin (from either non-diabetic or T2DM samples), cells from both non-diabetic and T2DM samples were distributed throughout the differentiation process (Fig. 4E).

Furthermore, variations in the expression of the five previously authenticated DEMGs during the differentiation of the three cell types were monitored. The DEMGs *CDKN1C* and *DLK1* of the neurons cluster exhibited a slightly upregulated expression pattern from neuronal cells to epithelial and smooth muscle cells, followed by a gradual decrease; whereas *SST* displayed a trend of progressive up-regulation and then down-regulation during the differentiation of neuronal cells into epithelial and smooth muscle cells, and the levels of this gene were almost equilibrated at the initial and terminal ends of the differentiation; for *TTR*, generally, it presented a decrease expression during the process of cell differentiation; the *SDC4* gene of the epithelial cells population manifested a relatively advanced expression at the end of differentiation (Additional file 1: Fig. S2).

## Analysis of T2DM-related DEMGs

**Fig. 5:**
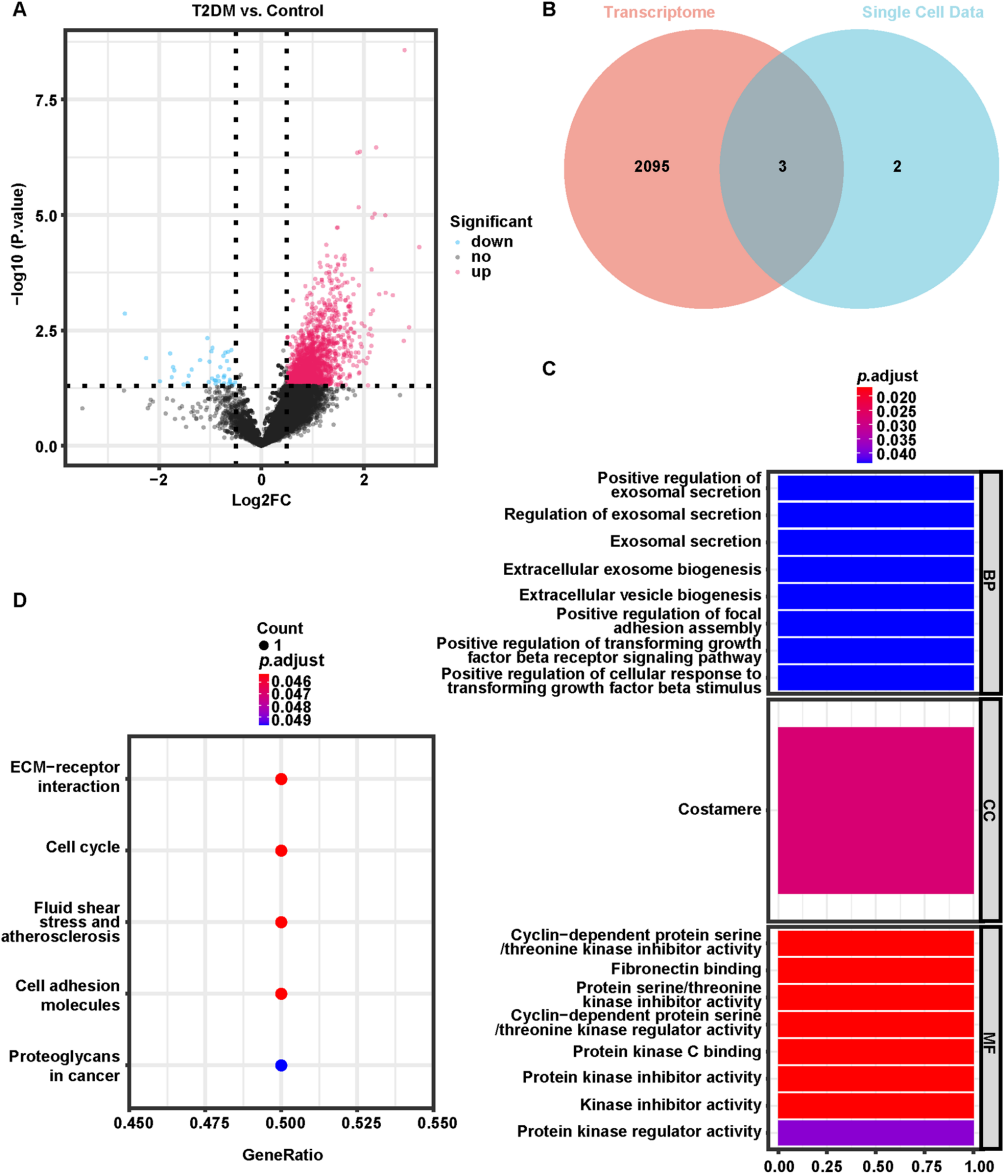
Analysis of T2DM-related DEMGs combining bulk RNA-seq and single cell data. **A **The volcano plot of GSE86468 dataset. **B** Identification of three T2DM-related DEGs by cross-tabulation analysis of bulk RNA-seq and single cell data. **C** GO analysis of T2DM-related DEMGs. **D** KEGG analysis of T2DM-related DEMGs

In the GSE86468 dataset, a total of 2098 DEGs linked to T2DM were authenticated by R package limma, using |log_2_ FC|> 0.5 and *P* < 0.05 as the significance threshold (Additional file 1: Table S10). Compared to non-diabetic patients (*n* = 15), 2058 genes were markedly upregulated in the T2DM (*n* = 9) population and 40 were downregulated in T2DM patients (Fig. 5A). The cross-tabulation analysis then indicated that *CDKN1C*, *DLK1* and *SDC4*, among the five previously characterized DEMGs, were also T2DM-related DEGs (Fig. 5B), and they were defined as T2DM-related DEMGs that may not only be implicated in the pathogenesis of T2DM but also exert crucial functions in the common cell clusters.

GO analysis highlighted that these T2DM-related DEMGs in the BP category were intimately affiliated with exosome secretion and its regulatory processes; meanwhile, the Notch signaling pathway, cell–matrix adhesion, and regulatory processes of T cell proliferation/activation were notably enrolled; in addition, these genes were inextricably linked to urological development (mesonephric development, mesonephric tubule/epithelial development, and ureteral bud development). Furthermore, these genes were potentially responsible for regulating the activity and binding of cell cycle proteins, protein kinase regulators, and protein kinase inhibitors in the costamere. The top 10 BP entries and all CC and MF entries are visualized in Fig. 5C, and the detailed GO analysis results are provided in Additional file 1: Table S11. In KEGG analysis, these T2DM-related DEMGs were principally engaged in pathways relevant to inflammatory responses, cell cycle, atherosclerosis, and proteoglycans in cancer (Fig. 5D; Additional file 1: Table S12).

## Assessment of the diagnostic value and clinical relevance of the T2DM-related DEMGs

**Fig. 6:**
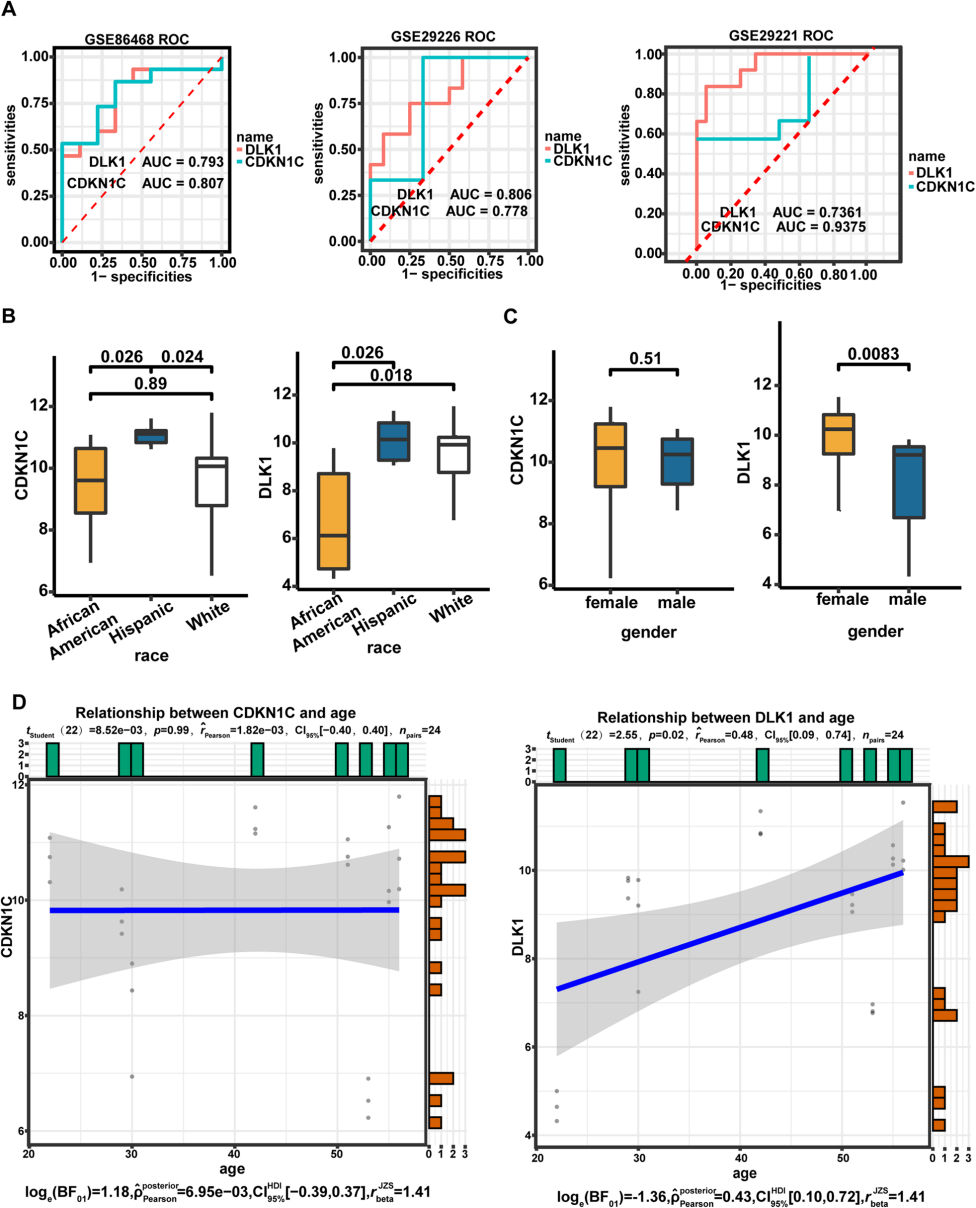
Diagnostic value and clinical relevance of T2DM-related DEMGs. **A** ROC curves for *CDKN1C* and *DLK1* in the GSE86468, GSE29226 and GSE29221 datasets. **B** The expression of *CDKN1C* and *DLK1* in the ethnic subgroups. **C** Differences between *CDKN1C* and *DLK1* in gender subgroups. **D** Relevance analysis between DEMGs and patient age

To appraise the usability of the three T2DM-related DEMGs in recognizing T2DM and non-diabetic samples, ROC curve analysis was performed based on their expression profiles extracted from the GSE86468 and GSE29226 datasets, respectively. The results revealed that *CDKN1C* and *DLK1* had favorable sample discrimination in both datasets (both AUC > 0.75; Fig. 6A) and considered as critical genes. The ROC results in GSE29221 confirmed the optimal predictive performances of these genes (both AUC > 0.7, Fig. 6A). Further, it was noticed that *CDKN1C* and *DLK1*, marker genes of neuronal cell clusters, were expressed differentially between non-diabetic and T2DM samples in epithelial cell clusters and smooth muscle cell clusters from GSE81608 and GSE86469 (Additional file 1: Fig. S3). Furthermore, using the Single Cell Expression Atlas database, it was also observed that the mRNA levels of two critical genes were significantly increased in type B pancreatic cells from T2DM patients (Additional file 1: Fig. S4A). On the other hand, the t-SNE map from the E-MTAB-5061 datasets indicated that the two critical genes expressed in both pancreatic endocrine cells and exocrine cells as well, such as acinar cells and ductal cells (Additional file 1: Fig. S4B).

Subsequently, the connection of *CDKN1C* and *DLK1* with the clinical traits of T2DM was evaluated. The results revealed that in the ethnic subgroups (African American, Hispanic, and White), both genes possessed the highest expression in the Hispanic population and the lowest expression in the African American population (Fig. 6B); in the gender subgroup, *DLK1* expression was considerably decreased in the male population compared with that in the female cohort, whereas *CDKN1C* was unremarkable with the gender of the patients (Fig. 6C). Relevance analysis confirmed that *DLK1* expression levels were positively linked to patient age (r = 0.48, *P* = 0.02), whereas *CDKN1C* was irrelevant to this trait (r = 1.82e-03, *P* = 0.99; Fig. 6D); moreover, non-association of both genes with patient BMI was evident (Additional file 1: Fig. S5).

## Exploration of the latent molecular mechanisms of the critical genes in the T2DM progression

**Fig. 7:**
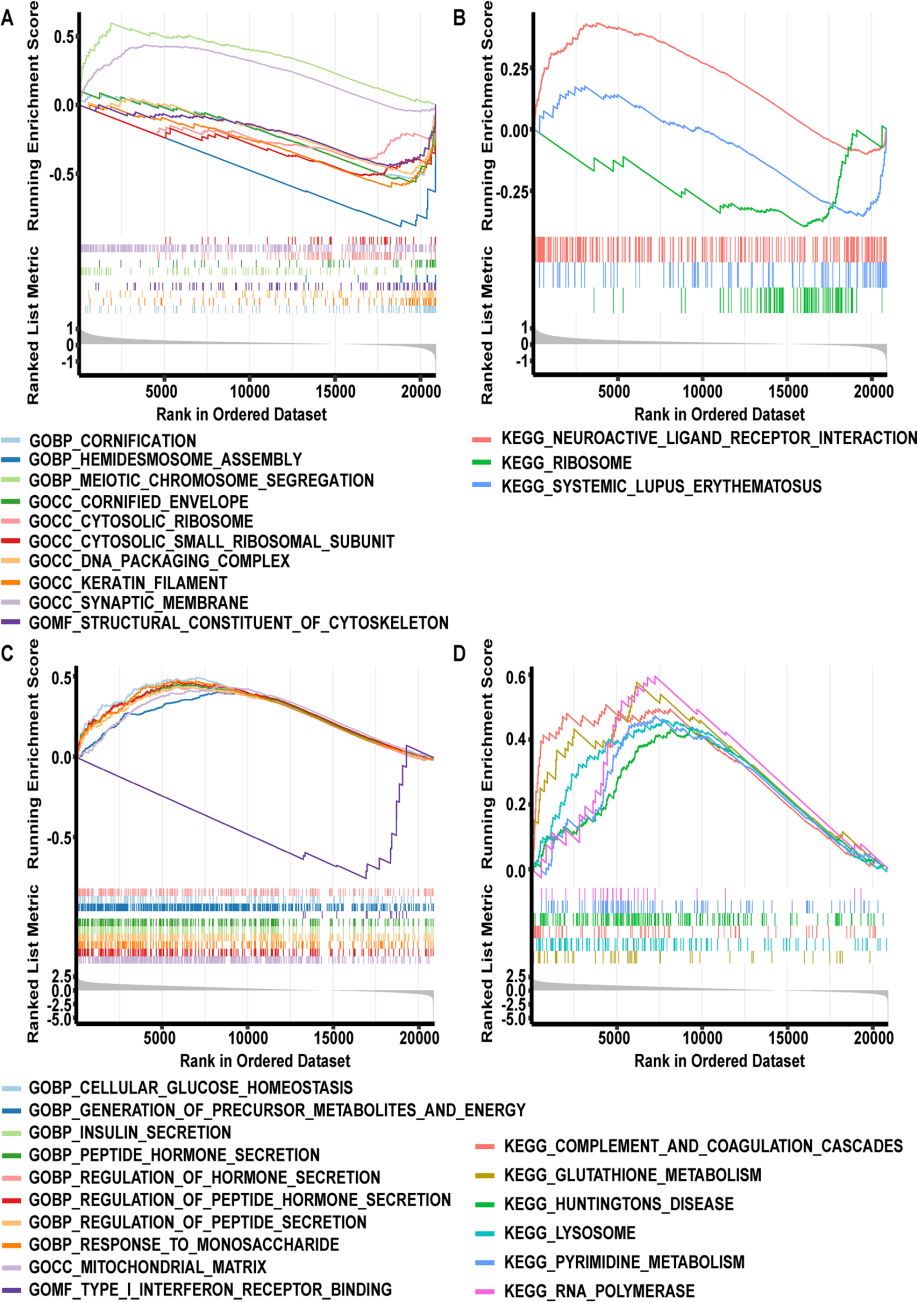
Latent molecular mechanisms of the critical genes in T2DM progression. **A** GO term for *CDKN1C* gene set enrichment analysis (GSEA). **B** KEGG term of gene set enrichment analysis (GSEA) for *CDKN1C*. **C** GO term for *DLK1* gene set enrichment analysis (GSEA). **D** KEGG term of *DLK1* gene set enrichment analysis (GSEA). The top portion of the plot shows the running enrichment score (ES) for the gene set as the analysis walks down the ranked list. The score at the peak of the plot is the ES for the gene set. The middle portion of the plot shows where the members of the gene set appear in the ranked list of genes. For a positive ES, the leading edge subset is the set of members that appear in the ranked list prior to the peak score. For a negative ES, it is the set of members that appear subsequent to the peak score. The bottom portion of the plot shows the value of the ranking metric when the list of ranked genes is moved down, measuring the correlation of gene with a phenotype and sorted using Signal2noise

An enrichment analysis using predefined gene sets revealed the possible molecular mechanisms of the critical genes in the T2DM process. For the critical gene *CDKN1C*, a total of 14 GO terms (Additional file 1: Table S13) and 3 KEGG pathways (Additional file 1: Table S14) were ensured. In Fig. 7A, B, the top 10 GO terms and all KEGG pathways were displayed, respectively. Collectively, *CDKN1C* was tightly integrated with keratinization, meiosis, and immune response. The analysis of GO terms for *DLK1* (Fig. 7C; Additional file 1: Table S15) hinted that *DLK1* might be directly tied to T2DM, and it was found that peptide/peptide hormone/insulin secretion and glucose/fatty acid processes were distinctly enriched; additionally, it was participating in responses to carbohydrates, metabolism of purine-containing compounds, and coagulation-related biological processes. The six KEGG pathways in which *DLK1* was engaged are displayed in Fig. 7D (Additional file 1: Table S16).

## Expression analysis of *CDKN1C *and* DLK1* by western blotting

**Fig. 8:**
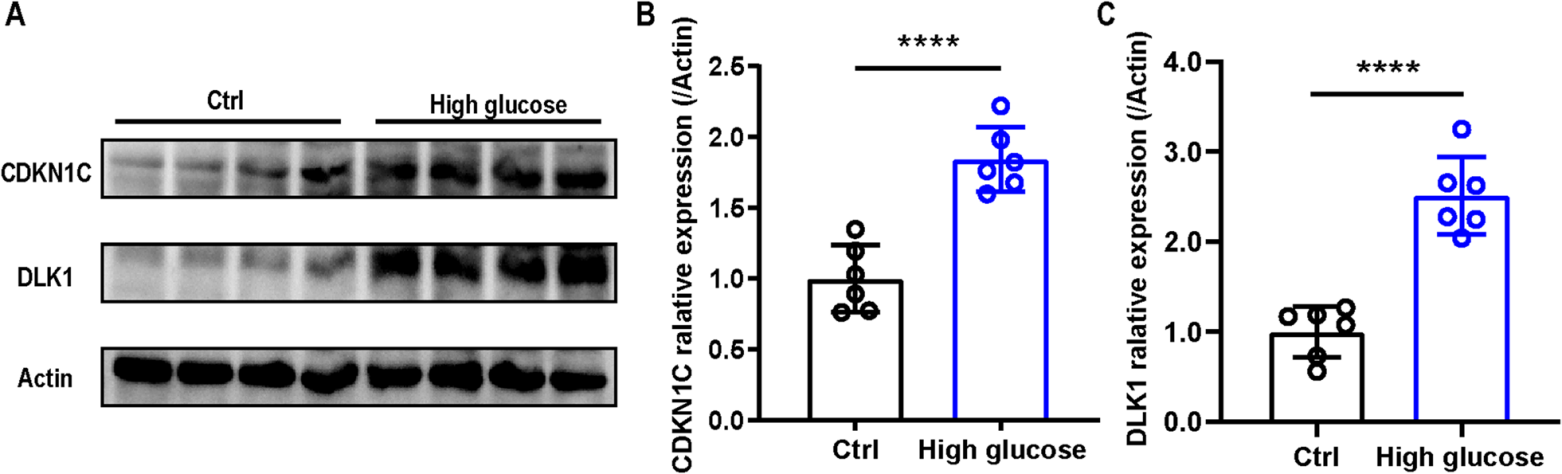
The alternation of *CDKN1C* and *DLK1* expression in primary pancreatic islet cells under different conditions. **A** Representative western blot of *CDKN1C* and *DLK1* in primary pancreatic islet cells treated with or without 30 mM glucose (HG) for 48 h. **B** Densitometric analysis of *CDKN1C* in primary pancreatic islet cells. **C** Densitometric analysis of *DLK1* in primary pancreatic islet cells

The expression of *CDKN1C* and *DLK1* was further confirmed in rat pancreatic islet cells. Compared with control group, *CDKN1C* and *DLK1* were upregulated after 30 mM glucose treatment for 48 h (Fig. 8).

## Projection of the potential compounds targeting the critical genes

Key gene–compound interactions were predicted using the PubChem database. A total of 98 potential compounds capable of affecting *CDKN1C* expression were predicted in the premise of Homo sapiens (Additional file 1: Table S17), of which, 11 compounds (lactic acid, hydroquinone, nickel, beta-naphthoflavone, disulfiram, valproic acid, vorinostat, sulforaphane, pirinixic acid, estradiol, and cytarabine) were reported to decrease the expression of *CDKN1C*. Of the 41 potential compounds predicted to affect *DLK1* expression (Additional file 1: Table S18), 21 compounds (2,2-bis(4-glycidyloxyphenyl)propane, benzo(a)pyrene, valproic acid, fluorouracil, entinostat, vorinostat, estradiol, sarin, tetrachlorodibenzodioxin, copper sulfate, doxorubicin, rosiglitazone, trichostatin a, tretinoin, thapsigargin, 4-(5-benzo(1,3)dioxol-5-yl-4-pyridin-2-yl-1h-imidazol-2-yl)benzamide, cyclosporine, sunitinib, panobinostat, dorsomorphin, and phenylmercuric acetate) were previously described to reduce *DLK1* expression. Valproic acid, vorinostat, and estradiol were discovered to simultaneously downregulate the expression of two critical genes.

## Discussion

The prevalence of diabetes mellitus is 9% worldwide, with type 2 diabetes (T2DM) accounting for 90% of all cases [9]. However, the underlying mechanisms of T2DM are poorly understood as yet. Although lifestyle and diet partially account for this health disparity, more than 100 robust association signals in T2DM have been identified in genome-wide association studies [10, 11], indicating the complex polygenic nature of T2DM. Thus, identification of the core genes may provide potential target for the treatment of T2DM. The pancreatic islet cells play an important role in the development of diabetes. The tremendous advance in transcriptomic analysis of single cells over the past five years has contributed to a better understanding of the role of islet cell-associated genes in the pathogenesis of diabetes. Given that scRNA-seq offers a unique perspective on the mechanism driving responses within and outside of the disease, it allows us to focus on cell alterations [6]. *CDKN1C* and *DLK1* of T2DM were identified by integrated analysis of scRNA-seq and bulk RNA-Seq in this study. They are further linked to the clinical characteristics of T2DM patients by internal and external validation. Additionally, the potential compounds targeting *CDKN1C* and *DLK1* in T2DM are forecasted and profiled. Our study provides a theoretical basis for investigating the mechanisms of T2DM progression and exploiting innovative therapies in the future.

In islet cells, endocrine cells have been shown to be identified by the expression of *INS* (beta cell), *GCG* (alpha cell), *SST* (delta cell), *PPY* (PP cell), *GHRL* (epsilon cell), while exocrine cells by *COL1A1* (stellate cells), *PRSS1* (acinar cells) and *KRT19* (ductal cells) [12–14]. However, the genetic alternation of others cells such as neurons, epithelial cells and smooth muscle cells have not received sufficient attention. Indeed, while the autonomic nervous system innervates beta, alpha, and delta cells in the islet of mouse, the sympathetic fibers preferentially innervate smooth muscle cells in the blood vessels and control hormone secretion in human islets by affecting local blood flow instead of modulating endocrine cell function directly [15]. In this study, cell cluster have been annotated by CellMarker database and neurons, epithelial cells and smooth muscle cells have been identified in both datasets. As expected, marker genes of neurons are mainly linked to T2DM-related processes, such as peptide/peptide hormone secretion and insulin secretion, suggesting a crucial role of neurons in control of hormone secretion. Actually, the degree of islets innervation is three times greater in patients with T2DM than in controls, and the increase in fibers is negatively correlated with insulin secretion and glucose tolerance [16]. Moreover, HMG20A, a chromatin factor involved in neuronal differentiation and maturation, has been reported for metabolism–insulin secretion coupling. What’s more, the functional consequence of T2DM-linked rs7119 SNP reducing HMG20A expression may lead to impaired beta cell mature function [17]. In addition to neuron alternations, marker genes in epithelial and smooth muscle cells are associated with immunity, wound healing, barrier function and CHD-related pathway, indicating that the inflammation and vascular injury may also be potential mechanism of T2DM in islet. Hence, accumulating evidences have shown that targeting islet inflammation may be an effective therapeutic strategy for T2MD patients [18].

*CDKN1C,* located on the human chromosomal band 11 p15.5, encodes the cyclin-dependent kinase inhibitor 1c (*p57, Kip2*), which is known as “a tumor suppressor gene” [19, 20]. It not only negatively regulates cell proliferation, but also directs the differentiation of certain select lineages, as well as maintains adult quiescent neural stem cell populations of adult quiescent [21]. Previous studies confirmed that *CDKN1C* played an important role in the neurogenesis, migration and morphology [20], of which overexpression could inhibit the proliferation of β-cells, leading to diabetes [22]. As indicated by the enrichment analysis, *CDKN1C* enriches in biological processes of keratinization, meiosis, and immune response. Expression levels of keratin 17 protein, which is involved in the keratinization pathway, are confirmed to be upregulated in T2DM [23]. Previous studies have shown that children of mothers with gestation diabetes are more likely to become obese and develop diabetes in adulthood, suggesting that meiosis may play a critical role in the development and progression of T2DM [24]. Dysregulation of adaptive immune cells may have relevance to T2DM and its comorbidities [25, 26]. Compared with control group, we found that the expression of *CDKN1C* was increased in primary pancreatic islet cells cultured with 30 mM glucose. In addition, the correlation with clinical characteristics has been validated with both internal and external datasets, suggesting that it may contribute to T2DM.

Delta-Like 1 Homolog (*DLK1*), a transmembrane protein to the Notch/Delta/Serrate family [27], is paternally expressed and belongs to a group of imprinted genes located on chromosome band 14q32 in humans and 12qF1 in mice [28]. It is expressed in many human tissues during embryonic development but is low in adults expression and is mostly restricted to (neuro)endocrine tissues and other immature stem/progenitor cells (notably hepatoblasts) [28], such as normal pituitary gland, spinal cord, pancreatic islet cells, adrenals, and Leydig cells [29]. Accumulating evidences demonstrate that *DLK1* plays an important role in energy metabolism [30] and exerts neuroendocrine effects [31]. In addition to inhibiting adipocyte differentiation [32], it also determines the cell fate of pancreatic islet cells and neurons [33, 34]. *DLK1* is involved in the differentiation of pancreatic ductal cells into β-cells, and promotes insulin synthesis and secretion by activating AKT signaling [35–37]. However, animal studies have shown that mice overexpressing *DLK1* are insulin resistant [38]. Our enrichment analysis also demonstrated that *DLK1* was significantly upregulated in pathways including cellular glucose homeostasis, insulin secretion, mitochondrial matrix and glutathione metabolism. There is a general consensus that T2DM is characterized by chronic hyperglycemia resulting from impaired insulin secretion [39] and altered glutathione metabolism [40], and mitochondrial dysfunction in adipose tissue partially participates in the pathogenesis of T2DM [41]. Compared with control group, the expression of *DLK1* was increased in primary pancreatic islet cells cultured with 30mM glucose. Combined its correlation with clinical characteristics validated by internal and external datasets, *DLK1* is expected to become a therapeutic target of T2DM [42]. However, *DLK1* is also associated with paternally inherited risk of type 1 diabetes, because the influence of SNP rs941576, which is in the imprinted region of chromosome 14q32.2 and at 105 kb downstream of *DLK1* [43]. Hence, further studies need to be performed to detect its role in different types of diabetes.

Notably, we predicted that 3 compounds could down-modulate *CDKN1C* and *DLK1* simultaneously. Valproic Acid (VPA), a histone deacetylase inhibitor, is widely applied in the treatment of bipolar disorder. In human embryonic stem cell (hESC)- based in vitro systems for developmental neurotoxicity and reproductive toxicity testing, VPA is used as a positive control compound to treat H9 hESCs. It can decrease the expression of *DLK1* in JRC and UKN1 system and the expression of *CDKN1C* in UKK system [44]. Vorinostat, another histone deacetylase inhibitor, also suppresses the expression of *CDKN1C* and *DLK* in UKN1 system by intervening H9 hESCs [45, 46]. Hence, VPA and vorinostat might be effective in reversing aforementioned islets deficits in T2DM, such as hormone secretion, inflammation and vascular injury, which remain to be verified in the future. In addition, *CDKN1C* and *DLK1* appear to be hormonally regulated, with the former decreasing after expose to estradiol in endometrial cancer cells [47], while the latter decreasing after treatment with estradiol in girls with anorexia nervosa or human uterine leiomyoma cells [48, 49]. Since the level of estradiol is correlated with age, especially for female, it is understandable why the expression of *DLK1* varies by gender and is positively correlated with age, albeit the underlying mechanism needs further investigation.

However, there were some limitations in this study. Further functional experiments based on knockout mice and large sample cohort studies are needed to confirm the regulatory mechanisms targeting these genes in various cell types of the human pancreas.

## Conclusion

In summary, we identified two core genes in T2DM though integrative analysis of two scRNA-seq dataset and a bulk RNA-seq dataset and validated their alternation in primary pancreatic islet cells cultured with or without high glucose. Moreover, the compounds associated with these key genes were predicted for further analysis in T2DM.

## Materials and methods

### Data source

The single-cell sequencing data (non-diabetic and T2DM patients) in our study were freely retrieved from the GEO database under accession numbers GSE86469 and GSE81608 which were generated using the same protocol: Smart-Seq RNA-sequencing method combined with by Fluidigm C1 system (SMARTer/C1) [50]. The GSE86469 dataset [14] contains 638 individual islet/other single-cell RNA-sequencing data from 5 non-diabetic and 3 T2DM samples (https://www.ncbi.nlm.nih.gov/geo/query/acc.cgi?acc=GSE86469). The GSE81608 dataset (https://www.ncbi.nlm.nih.gov/geo/query/acc.cgi?acc=GSE81608) encompasses a total of 1600 single-cell (islet) sequencing data from 12 non-diabetic samples and 5 T2DM samples [12]. The cell volume statistics for each type of sample comprised in the GSE86469 and GSE81608 datasets are presented in Table 2. And meanwhile, single-cell RNA-sequencing data from E-MTAB-5061 (contained the pancreatic tissue and islets from six non-diabetic samples and four T2DM samples) (https://www.ebi.ac.uk/biostudies/arrayexpress/studies/E-MTAB-5061?query=E-MTAB-5061) was downloaded from ArrayExpress (EBI) to explore the expression profiles of target genes in endocrine and exocrine cell types of the human pancreas [13]. Further, the GSE86468 and GSE29226 datasets were prepared to screen the target genes and estimate the diagnostic value of which supplemented by external validation set GSE29221. The GSE86468 dataset (platform: GPL18573) (https://www.ncbi.nlm.nih.gov/geo/query/acc.cgi?acc=GSE86468) entailed RNA-seq profiles of 24 individual islet/other bulk cell samples (obtained from 15 non-diabetic and 9 T2DM cadaveric organ donors) [14]. The GSE29226 dataset [51] (https://www.ncbi.nlm.nih.gov/geo/query/acc.cgi?acc=GSE29226) holds gene expression profiles based on 24 subcutaneous fat biopsies (three biological replicates and four technical replicates) from three T2DM patients and three non-diabetic patients (all female), which were produced with the Illumina HumanHT-12 v3 Expression BeadChip array (GPL6947 platform). The GSE29221 dataset (https://www.ncbi.nlm.nih.gov/geo/query/acc.cgi?acc=GSE29221) was generated on the platform of GPL6947 with the Illumina HumanHT-12 V3.0 expression beadchip and contained the transcription data from three T2DM and three non-diabetic individuals as per four times.

**Table 2:** Cell volume statistics for each type of sample

Dataset	Non-diabetic	T2D	All
GSE86469	380	258	638
GSE81608	651	949	1600

### Exclusion of the low-quality cells

The comprehensive analysis of single-cell sequencing data in this study was initiated through the ‘IntegrateData’ functions within R package Seurat (version 4.1.0) based on the GSE86469 and GSE81608 datasets, respectively. The exclusion criteria for low-quality cells in this study are as follows: (1) the number of detectable genes in a single cell was less than 100; (2) the proportion of mitochondrial genes in a single cell is ≥ 5%; and (3) the number of genes detected in a single cell is ≤ 3. Besides, genes that failed to detect expression in up to 3 single cells would be discarded. Eventually, in the GSE81608 dataset, we gained 1492 cells (622 derived from non-diabetic patients and 870 from T2DM samples) and 28134 genes. No low-quality cells were recognized in the GSE86469 dataset of 638 cells (non-diabetic and T2DM samples contained 380 and 258 cells, respectively), while 17280 genes from this dataset were captured in the follow-up analysis.

### Reduced-dimensional and categorization analysis

The ‘FindVariableFeatures’ function (vst method) was conducted to calculate feature variance on the standardized values after clipping to a maximum and select the high variability genes (top 2000) for downscaling on the filtered retained core cells. Next, the consistency of the sample distribution was monitored by principal component analysis (PCA). In this process, PCs with *P* < 0.05 were recognized for subsequently unsupervised cluster analysis (that is, K-means clustering algorithm) [52], where a single-cell resolution was combined with PCs in elbow plot to determine relevant sources of heterogeneity. Finally, the results of the unsupervised clustering analysis were visualized in nonlinear t-SNE [53] plots. The above analyses were executed in both GSE86469 and GSE81608 datasets.

### Notation of the cell subpopulation types

Following the findings of tSNE, the marker genes for each cell subpopulation were located in every dataset by utilizing the FindAllMarkers function. The parameters were set to min.pct = 0.5, logfc.threshold = 1, min.diff.pct = 0.3, and p_val < 0.05. Cell cluster phenotypes were determined by comparing marker genes of each cluster in the CellMarker database (SingleR package, version 1.6.1). The cell phenotypes which were advertised in both datasets were principally focused and denominated as the common cell clusters to ensure the robustness of the identified clusters [54, 55].

### Functional elucidation of the marker genes

The shared marker genes from each common cell cluster in both datasets (achieved by cross-tabulation analysis) were selected to implement a functional interpretation, aiming to preliminarily probe the latent mechanisms of each cell cluster in non-diabetic and T2DM. The analysis was undertaken in the R package clusterProfiler based on Gene Ontology (GO) and Kyoto Encyclopedia of Genes and Genomes (KEGG) databases. The enrichment criterion was established as *P* < 0.05. The cross-tabulations and the Venn diagrams presenting the results were executed in the online tool Jvenn, at the URL http://jvenn.toulouse.inra.fr/app/example.html.

### Pinpointing the aberrantly expressed marker genes from non-diabetic and T2DM cells within the common cell cluster

Differentially expressed genes (DEGs) between non-diabetic and T2DM cells were also characterized, respectively, in the common cell clusters of the corresponding datasets, and analyzed by the FindMarkers function of the R package Seurat. The threshold was set to |average log_2_-fold change (FC)|≥ 0.5 and *P* ≤ 0.05. Next, Differentially expressed marker genes (DEMGs) representing the corresponding common cell clusters were elicited by the crossover of marker genes from a single common cell cluster and DEGs within a single common cell cluster in both datasets.

### Putative time-series analysis of the common cell clusters

The differentiation trajectories and processes of the 3 common cell clusters were abstracted using Monocle [56]. Briefly, signature genes were screened in the common cell cluster using Seurat with the following criteria: (1) expression detected in no less than 10 cells; (2) |mean expression value|> 0.5; and (3) differential expression analysis q < 0.01. Then, pseudo-time-series analysis was undertaken by R package Monocle based on the authenticated signature genes. Eventually, a pseudo-time-series trajectory chart of the common cell phenotypes was visualized using the plot_cell_trajectory function.

### T2DM-related DEGs

The variability of gene expression levels between the T2DM group and the non-diabetic group was compared by R package edgeR in the GSE86468 dataset, with |log_2_ FC|> 0.5 and *P* < 0.05 set as the threshold for significant differences. Besides, intersection analysis was utilized to recognize DEMGs in the GSE86468-DEGs, defined as T2DM-related DEMGs, and R package clusterProfiler-based functional annotation was implemented for them (see 2.5 for details).

### ROC curve-based assessment of diagnostic benefit

The area under curve (AUC) of the receiver operating characteristic curve (ROC) was applied to appraise the usability of the ascertained T2DM-related DEMGs in diagnostic non-diabetic and T2DM samples. Briefly, the expression profiles of the T2DM-related DEMGs in the GSE86468 and GSE29226 datasets were matched, the ROC curves of the corresponding genes were plotted and the AUCs were calculated by R package pROC in both datasets [57]. Only genes with AUCs greater than 0.75 in both datasets were admitted in the ensuing analysis here. These genes were defined as “critical genes”. Further, the predictive performance of critical genes was validated by a external validation set (GSE29221).

### Enrichment analysis of pre-specified gene sets of the critical genes

Elucidating the hidden functions of the critical genes may facilitate the interpretation of the progression of T2DM. Here, the corresponding subsets (c5.go.v7.4.entrez.gmt; c2.cp.kegg.v7.4.entrez.gmt) were used as preset gene sets, which were freely available from the Molecular Signatures Database (MSigDB) [58, 59], URL: http://www.broadinstitute.org/msigdb. The samples were divided into high and low risk groups based on the median expression of the single key gene. Gene Set Enrichment Analysis (GSEA) was carried out by using R package clusterProfiler in the high and low risk groups in the T2DM cohort (GSE86468 dataset). Worthy terms/pathways were ascertained by |Normalized Enrichment Score (NES)|> 1, *P* < 0.05, and q < 0.25.

### Western blotting

Primary pancreatic islet cells (CP-R015) were purchased from Procell Life Science & Technology Co.,Ltd. They were cultured with or without 30 mM glucose (high glucose, HG) for 48 h. Protein concentrations were extracted using ice-cold RIPA buffer (Beyotime, Nantong, China) containing protease inhibitor and phosphatase inhibitor (Thermo Fisher Scientific, Waltham, MA, USA). A 20–30 μg protein was subjected to 10–15% SDS polyacrylamide gel electrophoresis and transferred onto polyvinylidene difluoride membranes (PVDF, Millipore, Bedford, MA, USA). The PVDF membranes were blocked in 5% skim milk for 1h and then incubated overnight at 4 ℃ with DLK1(sc-376755, Santa Cruz Biotechnology) or CDKN1C (sc-56341, Santa Cruz Biotechnology). After incubating with appropriate secondary antibodies for 2 h, the band densities were determined using Image J software (NIH, Maryland, USA) and normalized to each internal control.

### Forecasting and profiling of the potential compounds targeting the critical genes in T2DM

The interaction of compounds with critical genes in the PubChem database (https://pubchem.ncbi.nlm.nih.gov/) was pretested by implementing "drug–gene interactions" (default values were selected for parameters). In conjunction with the expression trend of critical genes in T2DM (relative to the non-diabetic group), compounds that could override this expression trend were suggested as the potential compounds for targeting the critical genes.

### Expression patterns of critical genes in single-cell-related datasets

Considering the significance of gene expressions in different cell types in T2MD and non-diabetic individuals, cells in GSE81608 and external E-MTAB-5061 datasets were further annotated with the help of islet tissue marker genes in the Single Cell Expression Atlas (https://www.ebi.ac.uk/gxa/sc/home) database to analysis the expressed difference of critical genes between T2DM group and non-diabetic group, including pancreatic A cell: TTR, GCG, CFC1, PCSK2, SLC38A4; pancreatic D cell: SST, LEPR, RBP4, SEC11C, HHEX; pancreatic PP cell: PPY, PPY2P, MEIS2, ETV1, ABCC9; pancreatic ductal cell: KRT19, CFTR, ANXA4, CLDN1, KRT8; pancreatic stellate cell: COL1A1, CALD1, SPARC, BGN, SERPINH1; type B pancreatic cell: INS, INS-IGF2, HADH, ADCYAP1, IAPP.

### Statistical analysis

The differences in the critical gene expression between samples (non-diabetic and T2DM samples) were detected by the Wilcoxon rank-sum test at *P* < 0.05, as were the detection methods and thresholds between different clinical characteristic subtypes (race and gender). The correlations between critical genes and patients' BMI and age were illuminated by Pearson correlation analysis, with remarkable correlations defined by | correlation coefficient (r)|> 0.3 and *P* < 0.05. The software packages and statistical analyses addressed in this study were exhaustively described in the corresponding positions. The difference of western blotting analysis was detected by t-test under the help of GraphPad Prism software (GraphPad Software, version 7.0). *P* value lower than 0.05 (*p* < 0.05) was considered as significant differences.

### Supplementary Information

**Additional file 1: Figure S1.** Visualization of cells distribution in GSE81608 and GSE86469 among clusters at different resolutions through K-means clustering algorithm. **A** GSE81608, the resolution is 0.7 was considered as a cutoff. **B** GSE86469, the resolution is 0.8 was considered as a cutoff.** Figure S2.** Expressions of the five DEMGs in the three cell types over trajectory time. Trend in expressions of five DEMGs during differentiation of three cell types over trajectory time.** Figure S3.** Expression of *CDKN1C* and *DLK1* in different cell clusters of non-diabetic and T2DM samples. **A** Expression of *CDKN1C* in different cell clusters of non-diabetic and T2DM samples. **B** Expression of *DLK1* in different cell clusters of non-diabetic and T2DM samples.** Figure S4.** Expression of *CDKN1C* and *DLK1* in the major endocrine clusters, acinar cell and ductal cell of non-diabetic and T2DM samples. **A** The expression level of *CDKN1C* and *DLK1* in major pancreatic cells of non-diabetic and T2DM patients. **B** The t-SNE plot of *CDKN1C* and *DLK1* expressed in endocrine cells and exocrine cells.** Figure S5.** Relevance analysis between DEMGs and patient age. **A** Relevance analysis between *CDKN1C* and patient age. **B** Relevance analysis between *DLK1* and patient age.

## Data Availability

The datasets [GSE86469, GSE81608, GSE86468, GSE29226 and GSE29221] for this study can be found in the GEO database (https://www.ncbi.nlm.nih.gov/geo/) and ArrayExpress (EBI) (https://www.ebi.ac.uk/biostudies/arrayexpress/studies/). The related code for present study could be found on GitHub at https://github.com/liyansong1987/code-for-manuscript.
